# Clinical, demographic, and laboratory characteristics of COVID -19 infection and risk of in-hospital mortality. A single center 4

**DOI:** 10.22088/cjim.13.0.211

**Published:** 2022

**Authors:** Fatemeh Nasseri Atashani, Elham Nasseri, Esmaeil Zeinali, Roya Zamani, Aliakbar Salahshouri, Mohsen Ghourchibeigi, Parnaz Heidari, Ali Koushan, Narges Naseri Atashani, Behzad Heidari

**Affiliations:** 1 Emergency Clinic, Shahriyar Hospital, Shahriyar, Karaj, Iran; 2 Department of Internal Medicine, Shahriyar Hospital, Karaj, Iran; 3 Sina Clinic Hospital, Babol, Iran; 4 Milad Hospital, Tehran, Iran; 5 Tehran University of Medical Sciences, Tehran, Iran; 6 Mobility Impairment Research Center, Babol University of Medical Sciences, Babol, Iran

**Keywords:** COVID-19, Mortality, Hospitalization, Elderly, Hypoxemia, Lymphopenia, Renal disease

## Abstract

**Background:**

Despite advances in preventive measures, COVID -19 spread and mortality is continuing due to delay in timely diagnosis. This problem is partly dependent on variations in disease characteristics, distribution of risk factors particularly comorbidities and demographic characteristics of patients. This study aimed to determine the clinical presentation and associated factors of mortality in patients hospitalized with COVID -19 infection.

**Methods:**

Patients were divided into survivor and deceased groups, and clinical and laboratory findings and factors associated with mortality between the two groups were compared by calculating odds ratio (OR) with 95% confidence interval (95% CI).

**Results:**

A total of 257 patients (female 45.1%) with a mean age of 59.8+15.7 years and a mean hospital stay of 4.89+3.57 days were studied. Diabetes, hypertension, cardiovascular disease and chronic renal disease (CRD) were found in 29.6%, 37.5%, 16.3% and 3.5% of all patients, respectively. Forty-one (16%) patients died. Factors such as age >50 years, coexisting CRD, serum creatinine > 2 mg/dl; SPO2 <70% lymphocytes < 20% during hospitalization were independently associated with mortality. The adjusted ORs (95% CI) were 10.08 (1.39-73); 4.51(1.15-17.61); 6 (1.14-31.5); 16.8(2.93-96.7); and 4.9(1.31-18.1), respectively. Most of the expected effective drugs were not associated with lower mortality.

**Conclusion:**

These results indicate a high in-hospital mortality rate in COVID -19 patients. Some mortality factors occurring during hospitalization were reversible and could be prevented by timely diagnosis and appropriate treatment.

Despite scientific advances related to SARS-CoV-2 infection and the development of vaccines, illness and death associated with COVID -19 infection continue to occur due to inadequate preventive measures or the emergence of more transmissible variants of SARS-CoV-2 or delays in diagnosis and vaccine preparation, distribution, and administration (1). Systemic inflammation in COVID -19 affects multiple organs, leading to disease progression and the development of complications that eventually lead to death in a significant proportion of patients with severe disease, especially in the elderly with concomitant disease (2). However, the course of infection varies depending on the characteristics of the disease, the patient's condition, and the presence of risk factors. Most Covid 19 patients show mild symptoms and a tolerant course. In a large study from China, 81% of patients with COVID -19 were classified as having mild disease, indicating no or mild pneumonia, while 14% had severe disease with hypoxemia and respiratory distress, and 5% had critical illness with respiratory failure, septic shock, and multiple organ failure (3, 4).

Several factors such as age, diabetes, hypertension, chronic kidney and lung disease were found to be associated with progression and severity of COVID -19 infection and mortality. Individuals with one or more underlying diseases such as diabetes, hypertension, and cardiovascular disease are expected to be at higher risk for severe disease, disease progression, and mortality (5, 6). In a large study, the overall mortality of COVID -19 was 2.3%, but the mortality in critical patients was 49% (3). 

A systematic review and meta-analysis of 7 eligible reviews found that 20.7% of patients presenting with COVID -19 infection had comorbidities such as hypertension, 9.6% cardiovascular disease, 9.55% diabetes, and 7% respiratory disease, and 9% cigarette smoking (7). The presence of these comorbidities, particularly in COVID -19 patients, especially the occurrence of end-organ damage, may differenty influence disease progression and thus predict disease outcome (8-10). A systematic review of 222 studies involving 281486 patients found severe COVID -19 disease in 23% and mortality in 6% of infected patients. Covid-19 severity was strongly associated with immunosuppression, diabetes and malignancy (11).

To date, no specific antiviral agent has been approved for the treatment of COVID -19 infection. Therefore, protective measures such as social distancing, avoidance of social gatherings, quarantine, and isolation remain the best effective preventive methods against the spread and acquisition of COVID -19. At the time of the pandemic, early diagnosis, especially detection of high-risk individuals, not only reduces the spread of the disease but may also prevent its progression. 

This issue emphasizes familiarity with early clinical features of COVID -19 infection and relevant risk factors. However, the clinical spectrum of COVID -19 ranges from asymptomatic or mild cases to severe disease with respiratory failure requiring admission to the intensive care unit (12). However, most patients present with fever, chills, myalgias, and respiratory symptoms such as dry cough and dyspnea. Gastrointestinal, musculoskeletal and neurological symptoms such as weakness and headache are also clinical features of COVID -19 infection in a large proportion of patients (13-15). The clinical presentation of COVID -19 is not limited to respiratory symptoms, as SARS-COV-2 infection can also affect other systems. A meta-analysis of 13 observational studies showed differences in the clinical presentation and outcomes of COVID -19 disease in different populations, which could be due to different epidemiological or demographic characteristics (16). 

The aim of the present study was to determine the pattern of clinical presentation and frequency of symptoms, as well as the prevalence of underlying chronic diseases in patients with COVID -19 infection. It also aimed to investigate their association with mortality in hospitalized patients.

## Methods

The participants of this retrospective cross-sectional study were selected consecutively according to the inclusion criteria among the patients admitted to Shahryar Hospital, Karaj Iran, for COVID -19 infection between September and December 2020. The diagnosis of COVID -19 was confirmed by polymerase chain reaction (PCR) test and/or the presence of typical radiographic findings on high-resolution CT scan of the lungs with compatible clinical symptoms. All patients who completed the treatment program during their hospitalization were included. Pregnant patients and those with acute cerebrovascular or cardiovascular disease and patients receiving hemodialysis were excluded.

All patients received a standard of care and underwent a complete clinical and laboratory examination at the time of inclusion. Data on demographic characteristics, clinical presentation, medical history, underlying chronic clinical conditions, laboratory findings, and treatment were collected from the patients' medical records.

 The study population was divided into a group of surviving patients and a group of deceased patients according to the outcome, and the two groups were compared in terms of clinical and laboratory characteristics and comorbidities to determine the factors associated with mortality. In statistical analysis, quantitative data were expressed as mean and standard deviation and categoric data as number and percentage in each category. The independent t-test was used to compare the quantitative variables. Chi-square test and Fisher's exact test with calculation of odds ratio (OR) and 95% confidence interval (95% CI) were used to determine the association. Independent association was determined using logistic regression analysis with calculation of adjusted OR. A two-sided p-value of less than 0.05 was considered statistically significant. SPSS software Version 18 was used for the analysis. The proposal for this study was approved by the local ethics committee of Shahryar Hospital, Kraj, Iran.

## Results

A total of 257 (female, 45.1%) patients were studied. The mean age was 59.8+15.7 years and the mean length of hospital stay was 4.89+3.57 days. Thirty-nine patients were admitted to the ICU and 41 (16%) patients died (21 males, 20 females). The mean age of patients who died was significantly higher (67.6+14.8 vs. 58.3+15.4, P= 0.001) (table 1).

**Table 1:** Clinical and demographic chatacteristics of the study population according to outcome

**Characteristics**	**Total sample** **n= 257**	**Surviving group** **N=216**	**Deceased group** **n=41**	**p value** ^¥^
Age, mean + SD. years	59.8+15.7	58.3+15.4	67.6+14.8	0.001
Age group, no(%) < 50 50 - 69 > 70	67 (26.1) 121(47.1) 69 (26.8)	62(28.7) 105(48.6) 49(22.7)	5 (12.2) 16(39) 20(48.8)	- 0.9 0.002 0.001
Sex, no (%) Men Women	141 (54.9) 116(45.1)	120 (55.6) 96(44.4)	21(51.2) 20( 48.8)	0.80 0.36
Duration of hospitalization, days	4.89+3.57	4.9+3.4	4.9+4.2	0.24
ICU admission, no (%)	39 (15.1)	28 (68.2)	11(26.8)	0.001
Clinical presentation , no(%) Fever and chills Respiratory symptoms Upper Lower Headache Musculoskeletal symptoms Gastrointestinal symptoms	136 (53.) 14(5.4) 223(86.6) 24 (9.5) 125 (48.6) 70 (27,2)	121(55.9) 14(6.7) 192 (88.9) 23 (10.8) 112 (51.8) 57(26.4)	15(37.5) 0 31(75.6) 1 (2.4) 13 (31.7) 13(31.7)	0.024 0.099 0.044 0.020 0.031 0.069
Other symptoms (Chest pain,weakness, neurologic symptoms, impaired level of consciousness)	72 (28.1)	58 (27.1)	14 (34.1)	0.24
Comorbidities, no (%) Cardiovascular disease Diabetes Hypertension Chronic lung diseases Cancers Neurovascular diseases Chronic renal diseases Inflammatory rheumatic diseases	42 (16.3) 76 (29.6) 96 (37.5) 21 (8.2) 6(2.3) 6 (2.3) 9 (3.5) 5 (2.1)	35(16.2) 63 (29.2) 76 (35.3) 18 (8.3) 5 (2.3) 4 (1.9) 5 (2.3) 4 (2)	7(17) 13 (31.7) 20 (48.8) 3 (7.3) 2 (4.3) 2 (4.8) 4 (9.8) 1 (2.4)	0.17 0.72 0.21 0.24 0.82 0.64 0.04 0.98
History of medication, no (%) Corticosteroids Immunosuppressive drugs Statins Aspirin Antimalarial drugs	4 (1.6) 4 (1.6) 37 (14.4) 39( 15.4) 4 (1.6)	3(1.3) 3 (1.3) 32 (14.9) 34(15.9) 4 (1.9)	1 (2.4) 1(2.4) 5(12.2) 5 (12) 0	0.46 0.46 0.89 0.87 NA^&^

^¥ ^Comparison of survived and decease patients ^&^ Not applicabe

The type and frequency of clinical symptoms are shown in table 1. Upper and lower respiratory symptoms such as sore throat, rhinorrhea, cough, dyspnea, chills and fever were the most common clinical symptoms in the study group. Musculoskeletal symptoms such as myalgias and bone pain were noted in almost half of the patients, and gastrointestinal symptoms in about a quarter of the patients. A proportion of patients had various symptoms such as chest pain, weakness, neurological symptoms and impaired consciousness. The incidence of concomitant diseases such as cardiovascular disease, hypertension, cerebrovascular disease, diabetes and other chronic diseases are shown in table 1. The proportion of these diseases, with the exception of chronic kidney disease and history of medication use, did not differ between deceased and surviving patients. The results of the laboratory tests are listed in table 2 regarding patient outcomes. Laboratory manifestations such as leukocytosis, lymphopenia, hypoxemia defined as peripheral oxygen saturation (SPO2) of < 90%, and impaired renal function defined as serum creatinine concentration >2mg /dl were significantly associated with mortality.

**Table 2:** Laboratory characteristics of hospitalized patients with COVID-19 infection according to outcomes

Characteristics	Surviving group	Deceased group	P value
White cell count, per microliter, mean + SD	8187 + 6961	10517 + 4668	0.043
Leukocytosis ^¥^, no (%)	49 ( 22.7)	21(52.5)	0.001
Lymphocytes %	19.5 + 10.1	12.5 + 9	0.001
Lymphopenia ^£^, no (%)	54.6%	80%	0.001
Platelet count, per microliter of blood × 1000.	207 + 78	201 + 85	0.70
Hemoglobin, gr/dl	13.1 + 2.2	13.1+ 2	0.97
ALT, IU/L	37.9 + 32	52.1 + 56	0.20
AST IU/L	47.3 + 39	97.1 + 96	0.012
Alkalain phosphatasis, IU/L	211 + 118	227 + 102	0.59
ESR, mm/h	61 + 72	51 + 32	0.26
PT, second	15.25 + 2.5	15.75 + 2.1	0.28
PTT,second	30.97 + 3.6	31.2 + 4.7	0.85
INR	1.29 + 1.08	1.23 + 0.22	0.61
Amylase, U/L	67 + 47.8	164 + 399	0.30
Total billirobin,mg/dl	0.73 + 0.37	0.81 + 0.46	0.51
Direct billirobin, mg/dl	0.22 + 0.16	0.30 + 0.26	0.26
Troponin, ng/ml	188 + 2	37 + 1.9	0.28
Creatinine, mg/dl	1.28 + 0.51	1.63 + 0.74	0.006
Renal function impairment ^&^, no (%)	13(6.2)	14 (34.1)	0.001
BUN, mg/dl	42 + 24	67 + 36	0.001
SPO2 % ^$^, mean + SD Moderate hypoxemia (70-89%), no (%) Severe hypoxemia (< 70%)	87.8 + 9.9 84(38.8) 13(6)	76 .7 + 14.5 14(34.1) 19(46.3)	0.001 0.001 0.001
LDH, U/L	598 + 237	1025 + 1214	0.001
Elevated LDH ^€^, no (%)	82(62.1)	20 (83.3)	0.034

^&^ > 10.000 per microliter

^£^ < 20 %

^& ^Serum creatinine > 2 mg /dl

^$ ^Peripheral oxygen saturation (SPO2) %

INR (international normalized ratio).

PTT = Partial thromboplastin time

ESR = Erythrocyte sedimentation rate > 10.000 per microliter

^€^ > 1000 U/L

Table 3 lists various drugs used to treat the patients. Interferon beta and anticoagulants were used in most patients and dexamethasone in 215 (84%) patients. Antiviral agents such as favipiravir and remdesivir were administered in 50 (19.5%) and 77 (30%) patients, respectively. The association between treatment and hospital mortality is shown in table 3. The proportion of mortality in patients treated with ivermectin, remdesivir, dexamethasone, and prednisolone was significantly higher than in patients who did not receive these drugs, suggesting an association between treatment and mortality. Twenty-two of 77 (28.6%) remdesivir-treated patients died, compared with 19 of 179 (10.6%) non-treated patients (P= 0.001). A subgroup analysis of remdesivir-treated and non-treated patients showed similar results for all risk factors except lymphopenia and hypoxemia, which were significantly more common in the treated subgroup (72% vs. 55.7%, P=0.006) and (20.8% vs. 9%, P= 0.001), respectively. In contrast, treatment with doxycycline was significantly associated with lower mortality than patients not treated with this drug (12.5% vs. 26.6%, P=0.009). Similarly, there was a trend toward lower mortality in patients treated with hydroxychloroquine and diphenhydramine, and a trend toward higher mortality in patients receiving aspirin. 

There were no significant therapeutic differences between treated and untreated subgroups for the other treatment groups, particularly favipiravir and interferon. The independent association between treatment and COVID -19 outcome, after adjustment for demographic, clinical, and laboratory factors, is shown in table 4.

In univariable analysis, age > 50 years, coexisting chronic kidney disease, presence of lower respiratory symptoms, worsening renal function during hospitalization, leukocytosis, lymphopenia, and hypoxemia were associated with increased risk of death. In addition, treatment of patients with remdesivir, ivermectin, dexamethasone, and prednisolone was significantly associated with an increased risk of death, and treatment with doxycycline was significantly associated with a decreased risk of death (table 4).

The results of multivariate logistic regression analysis after adjustment for all covariates (tables 1 and 2) showed that age 50-69 years was associated with a 10.08 (1.39-73) times increased risk of death and age > 70 years was associated with a 7.75 (1.89-31.8) times increased risk of death. Among laboratory tests, impaired renal function, lymphopenia, hypoxemia, especially SPO2 of <70% were significantly associated with mortality. Treatment of patients did not reduce the risk of mortality, but in contrast, remdesivir therapy was a significant and independent risk factor for mortality with an odds ratio of 14.50 (3.33- 66). 

Other drugs such as nonsteroidal anti-inflammatory drugs, vitamin D, C, pyridoxine, atorvastatin, hydroxychloroquine, prednisolone and dexamethasone did not affect the COVID -19 outcome . Similarly, the significant increase in mortality risk due to ivermectin and the significant protective effect of doxycycline declined to a non-significant levels and the non-significant level of hydroxychloroquine also remained unchanged. Nevertheless, after controlling the effect of all covariates, there was a trend towards a non-significantly reduced risk of mortality in patients treated with favipiravir.

**Table 3:** Types of medications and proportions of mortality in treated versus untreated patients in COVID-19 infection*

**Medications, no of patients treated (%)**	**Mortality, no (%)**		
	**Treated**	**Untreated**	**p value** ^&^
Favipiravir, 50 (19.5)	11(22)	30(14.6)	0.19
Aspirin ,108(42.2)	22(20.4)	19(12.8)	0.07
Hydroxychloroquine , 86(35.6)	9(10.5)	32(18.8)	0.059
Ivermectin (34(13.3)	10(29.4)	31(14)	0.026
Remdesivir, 77( 30)	22(28.6)	10(10.6)	0.001
Atorvastatin, 146 (57)	23(15.8)	18(16.4)	0.51
Zinc , 68 (26.5)	12(17.6)	29(15.4.	0.40
Vitamin C 152 (59.4)	26 17.1	29(15.4)	0.35
Vitamin D3 ,89(34.7) Diphenhydramin, 105 (41) Vitamin B6, 54 (21)	13(14.6 12 (11.4) 10(18.5)	28(16.8) 29(19.2) 31 (15.4)	0.39 0.09 0.58
Doxycycline,192 (75)	24(12.5)	17(26.6)	0.009
Cholchicine, 95 (37.1)	12(12.6.	29(18)	0.16
Bromhexine,91(35.5)	14(15.4)	27(16.4)	0.49
Famotidine ,97( 37.8)	28 (14.2)	13(22)	0.11
Interferon beta, 238(93)	29(14.7)	12(20.3)	0.20
Anticoagulants , 238 (93)	38(16)	3(16.7)	0.57
Dexamethason ,215 (84)	39 (18.1)	2 (4.9)	0.021
prednisolone , 25 (9.8) Naproxen,109 (42.5)	11(44) 16(14.7)	30(13.3) 25(17)	0.001 0.61

^*^ Data available for 256 patients

^&^ Conparison of treated and untreated patients using chi-square test

**Table 4:** Univariable and mulativariable logistic regression analysis to determine the associated factors of mortality in hospitalized patients with COVID-19 infection with calculation of odds ratio (OR) and adjusted OR (AOR) after controlling for all covariates with 95 % confidence interval (95 % CI)#

Covariates	Univariable regression		Multivariable regression	
	OR (95% CI)	P value	A OR (95% CI)	Pvalue
Age groups < 50 50 -69 > 70	1 5.06(1.77-14.45) 2.67(1.27-14.45)	0.002 0.009	1 10.08 (1.39-73) 7.75( 1.89- 31.8)	0.022^*^ 0.004^*^
Sex , Male vs. female	0.84(0.43-1.69)	0.11	3.31 (0.96-11.4	0.057
Fever and chills , Yes vs. No Cardiovascular disease Diabetes Hypertension Chronic lung diseases Cerebrovascular disease Chronic renal disease	0.52(0.25-1.068) 1.047(0.43-2.55) 1.10(0.53-2.27) 1.70 (0.86- 3.34) 0.85 (0.24 - 3.04) 2.67 (0.47-15.1) 4.54( 1.16-14.28)	0.053 0.17 0.45 0.84 0.55 0.25 0.018	1.92(0.93-3.94) 2.18 (0.37- 12.8 1.81 ( 0.47- 6.95) 0.59 (0.17-1.97) 1.16(0.32-4.16) 0.37(0.066-2.10) 4.51 (1.15 - 17.61)	0.075 0.38 0.38 0.39 0.80 0.28 0.030
Respiratory symptoms. Ys vs.No	0.41 (0.16- 1.07)	0.066	2.4(0.93-6.24)	0.07
Musculoskeletal symptoms, Yes vs. No	0.51(0.24- 1.08)	0.056	1.o4(0.92-4.09)	0.08
Renal function impairment,^ & ^ Yes vs. No	3.32 (1.62-6.82)	0.001	6(1.14-31.5)	0.034
Leukocytosis, yes vs.no Lymphopenia, yes vs. no	3.75(1.86- 7.55) 4.2(1.72- 10.67)	0.001 0.001	1.69( 0.52-5.51 4.9 (1.31 18.1)	0.38 0.018
SPO2 , 90-100% 70-89% <70%	1 8,7(3.55-216) 21.5 (7.9 -58.9	0.001 0.001	1 8 (1.65-39.5 16.8( 2.93-96.7)	0.01^₩^ 0.002^₩^
Elevated LDH^¥^, Yes vs. No	5.6 (1.85-1.08)	0.001	3.04(0.96-9.43)	0.053
Remdesivir, Yes vs. No	3.36 (1.69-6.68)	0.001	14.50 ( 3.33- 66)	0.001
Doxycycline, Yes vs. No	0.38 (0.19- 0.78)	0.007	1.30( 0.31-5.43)	0.71
Ivermectin, Yes vs. No	2.56(1.12-5.88)	0.017	0.85(0.18- 3.85)	0.83
Dexamethasone, Yes vs. No	4.2(0.97- 18.1)	0.057	1.36( 0.16-11.21)	0.77
Anticoagulants, Yes vs. No	0.82(0.22- 3.02)	0.77	1.21(0.33-4.46)	0.77
Interferone beta, Yes vs. No	0.66 (0.31-1.39)	0.27	0.78(0.17-3.53)	0.74
Famotidine, Yes vs. No	0.57 (0.27- 1.19)	0.13	2.97(0.81-10.91)	0.10
Bromhexine, Yes vs.no	0.92(0.45 - 1.86)	0.82	0.93(0.26-3.23)	0.90
Diphenhydramine,yes vs.no	0.54(0.26-1.12)	0.095	2.92(0.68-12.3)	0.14
Colchicine, Yes vs no	0.65(0.31-1.35)	0.24	2.42(0.62-9.43)	0.20
Vitamin D, Yes vs.no	0.84(0.41-1.72)	0.63	0.76(0.19-2.91)	0.69
Pyridoxine, Yes vs.no	1.24(0.56-2.73)	0.58	0.83(0.17-4.15)	0.82
Vitamin C , Yes vs no	1.19(0.60-2.39)	0.61	0.70(0.18- 2.66)	0.60
Zinc,yes Yes.no	1.16(0.55-2.4)	0.68	1.28(0.34-4.85)	0.70
Atorvastatin, Yes vs no	0.94(0.48- 1.85)	0.87	3.2(0.72-14.3)	0.12
Hydroxychloroquine, Yes vs. no	0.50(0.22-1.11)	0.08	1.23(0.34- 4.39)	0.74
Aspirin, Yes vs. no	1.73(0.88-3.4)	0.621	0.36(0.10-1.33)	0.12
Naproxen, Yes vs. No	0.84(0.42-1.66)	0.19	2.01(0.50-7.97)	0.32
Favapiravir, Yes vs. No	1.65(0.76-3.58)	0.14	0.28(0.078-1.02)	0.054
Prednisolone , Yes vs .No	5.23(2.17-12.6)	0.001	0.26(0.053-1.29)	0.10

^#^ Data available for 256 patients

^*^ Compared to < 50 years

^₩^Compared to SPO2 > 90%s

^¥^Greater than > 1000 U/L

^ & ^Creatinine > 2 mg /dl

^¥^Greater than > 1000 U/L

## Discussion

The results of this study indicated a mortality rate of 16% in hospitalized patients with COVID-19 infection. Patients aged > 50 years in particular age group of 50-69 years had significantly higher risk of mortality. Male- sex was marginally at higher risk of mortality compared to females. The rate of mortality in patients of this study is in agreement with total mortality of COVID-19 infection as observed in a review of 33 studies by Lim et al. (17). Similarly, higher proportion of death rate in older patients particularly > 50 years is consistent with the results of a meta-analysis by Bonanad et al. which found a mortality rate of < 1 % in age group < 50 years and 16.9% in 70-79 years and 24.4% in > 80 years(18).

In this study, none of the clinical symptoms were predictors of COVID-19 outcome, but occurrence of some laboratory abnormalities at the time of hospitalization including renal function impairment indicating serum creatinine levels > 2 mg /dl , lymphopenia and hypoxemia especially SPO2 < 70% were associated with higher mortality. Among the several comorbidities, only coexisting chronic renal disease was associated with higher mortality, whereas other comorbidities especially diabetes, cardiovascular disease and hypertension were not associated with mortality. This may be attributed to high percentage of diabetes, hypertension and cardiovascular disease in the survivor group reflecting high prevalence of these conditions in the general population or inadequate sample size (table1) and (15).

Similar associations were observed in other studies (15, 18-21). A systematic review and meta-analysis found a significantly higher mortality rate among COVID-19 patients with chronic renal diseases by OR = 5.81 (95% CI 3.78–8.94, p< 0.00001) (22). In the present study, the results of treatment on COVID-19 outcome differed from previous studies (23,-29). Among several medications which were used for the treatment of COVID-19 infection, only favapiravir was marginally associated with reduced mortality. However this drug was administered only in 50 of 256 (19.5%) patients. A study with a larger sample size may be associated with a better outcome. A systematic review and meta-analysis revealed a significantly higher clinical and radiological improvement in favapiravirr-treated versus control group who received a standard of care (RR =1.29 (95%CI, 1.08–1.54), but treatment with this drug had no significant effect on viral clearance or lower oxygen support (30). Treatment with other antiviral, anti-inflammatory, immunomodulating drugs and vitamins had also no effect on disease outcome as compared with untreated groups (table 3) except remdesivir which was associated with an unexpectedly higher mortality as compared with non-treated patients (OR= 14.50 ( 95 % CI, 3.33- 66). However, increased mortality in remdesivir group may be attributed to higher prevalence of lymphopenia and hypoxemia in this group. It is also possible that remdesivir was used in patients with more severe COVID-19 disease. Nevertheless, only a small number of patients received remdesivir and so the results are not adequately powered. It should be noted that the results of treatment with remdesivir in COVID-19 infection are conflicting across studies (31-33). A large study of 1062 patients who received remdesivir or placebo, the median recovery time in remdesivir group was shorter and clinical improvement was higher and evidence of respiratory infection was lower as compared to placebo (33). In contrast, the results of 2 RCTs demonstrated that treatment with remdesivir was not associated with significant clinical improvement as compared with placebo or a standard of care (31, 32). Conflicting results could be attributed to severity of COVID-19, epidemiological characteristics of the study patients, dosage and duration of treatment and the time of initiation of treatment. In a study by Brigel et al. (33) initiation of treatment at day 15 was associated with mortality of 6.7% in remdesivir and 11.9% in the placebo group, whereas the beginning of treatment at day 29 was associated with mortality of 11.4% and 15.2% in the remdesivir and placebo groups, respectively (HR=0.73, 0.52-1.03) (33).

Treatment of COVID-19 with ivermectin and colchicine in earlier studies was associated with decreased risk of mortality (3, 34, 35), but in the present study, both drugs had no effect on disease outcome. This may be due to small sample size or lack of a control group. Only 13.3% and 37.1% of patients received ivermectin or colchicine, respectively. Similarly bromhexine administration in our patients was not associated with change in COVID-19 outcome, whereas, in one study, the early administration of bromhexine was associated with a reduced rate of ICU admission, intubation and mortality (36). A non-significant association between corticosteroid therapy and COVID-19 mortality rate as shown in this study was also found in another study (37). However, a systematic review and meta-analysis revealed lower all-cause mortality in patients treated with corticosteroids as compared with patients who received only usual care or placebo (38). In the present study, most of the study patients received dexamethasone and the number of patients who did not receive dexamethasone were very small, so the results regarding the efficacy of dexamethasone are not powered. 

In the present study, no association was seen between hydroxychloroquine (HCQ) therapy and COVID -19 outcome. These findings are consistent with the results of another study which compared mortality of COVID-19 patients in HCQ users with or without azithromycine (39). However, in another study with large sample size, treatment with HCQ alone or in combination with azithromycin was associated with a significantly decreased risk of mortality (40). Lack of association between HCQ therapy and COVID-19 outcome in this study can also be explained by insufficient sample size and absence of a control group. In -hospital mortality rate in statin treated and non-treated patients was 15.8% and 16.4%, respectively indicating no association between statin therapy with mortality. These findings are in contrast with the results of a systematic review and meta-analysis of 13 studies with a total of 52,122 patients which has found a significantly reduced risk of in-hospital mortality with statin therapy (41). Treatment with aspirin and naproxen in these patients had no effect on COVID-19 outcome. 

The results of studies regarding the association between NSAIDs and COVID-19 mortality are controversial (42-44). While, the routine use of NSAIDs prior to hospitalization had no effect on mortality, but in patients with osteoarthritis /rheumatoid arthritis, COVID-related death in current users was lower than nonusers (43). Similarly in another study, aspirin use was independently associated with decreased risk of mortality, ICU admission and mechanical ventilation (44).

Vitamin supplementation including vitamin C, D and pyridoxine which had been administered in small proportions of patients of this study had no effect on COVID-19 outcome. An association between vitamin D deficiency/ insufficiency with severity of COVID-19 as well as hospitalization and mortality was shown (45). Although a small sample size of supplementation and lack of a control group can explain the discrepancies. Nevertheless, serum vitamin D level in patients of this study has not been assessed to detect high risk patients. The impact of treatment with famotidine on COVID-19 outcome in a small proportion of patients of this study was in agreement with the results of a systematic review and meta-analysis of 5 studies which found no significant effect of famotidine in reducing COVID-19 severity, death (46). Doxycycline exerts an antiviral and anti-inflammatory activity with dampening effect on cytokine storm in preventing lung damages (47). Early treatment in nonhospital setting resulted in early clinical recovery, decreased hospitalization and mortality (48). Doxycycline was administered in 75 patients of this study and was associated with lower mortality rate. However, mortality difference between treated and untreated group decreased to a nonsignificant level after adjustment for confounding factors. 

Overall, in the present study, the prevalence and the associated factors of mortality in COVID-19 infection were comparable with other studies, but the association between response to medical treatment and mortality rate is different from previous studies.

This study has several limitations including inadequate sample size, absence of a control group for comparison, lack of data regarding the time of beginning treatment, drug dosage, duration of treatment and severity of COVID-19 particularly the extent of lung involvement at the time of hospitalzation. These factors can differently affect the results and lead to biases. Nevertheless, the study has some strengths concerning patient selection in which all participants were dervied from a population with homogeneous characteristics, as well as similarity in treatment modality.Therefore, it is expected that the confounding factors being distributed similarly between the surviving and deceased groups and the results are likely biased minimallys. 

In conclusion, the results of this study indicate a mortality rate of 16% in hospitalized patients with COVID-19 infection. Age > 50 years, coexisting chronic renal disease, hypoxemia, lymphopenia and worsening of renal function during hospitalization are independent predictors of mortality. Most of the expected effective drugs were not associated with lower mortality.

## References

[1] Li M, Zhang Z, Cao W (2020). Identifying novel factors associated with COVID-19 transmission and fatality using the machine learning approach. Sci Total Environ.

[2] Inciardi RM, Solomon SD, Ridker PM, Metra M (2020). Coronavirus 2019 Disease (COVID-19), systemic inflammation, and cardiovascular disease. J Am Heart Assoc.

[3] Wu Z, McGoogan JM (2020). Characteristics of and important lessons from the coronavirus disease 2019 (COVID-19) outbreak in China: summary of a report of 72 314 cases from the Chinese center for disease control and prevention. JAMA.

[4] Davies NG, Jarvis CI (2021). Increased mortality in community-tested cases of SARS-CoV-2 lineage B.1.1.7..

[5] Zhou F, Yu T, Du R (2020). Clinical course and risk factors for mortality of adult inpatients with COVID-19 in Wuhan, China: a retrospective cohort study. Lancet.

[6] Gu T, Mack JA, Salvatore M (2020). Characteristics associated with racial/ethnic disparities in COVID-19 outcomes in an academic health care system. JAMA Netw Open.

[7] Hatmi ZN (2021). A systematic review of systematic reviews on the covid-19 pandemic. SN Compr Clin Med.

[8] Tian W, Jiang W, Yao J, e al (2020). Predictors of mortality in hospitalized COVID-19 patients: A systematic review and meta-analysis. J Med Virol.

[9] Emami A, Javanmardi F, Pirbonyeh N, Akbari A (2020). Prevalence of underlying diseases in hospitalized patients with COVID-19: a systematic review and meta-analysis. Arch Acad Emerg Med.

[10] Böger B, Fachi MM, Vilhena RO (2021). Systematic review with meta-analysis of the accuracy of diagnostic tests for COVID-19. Am J Infect Control.

[11] Li J, Huang DQ, Zou B (2021). Epidemiology of COVID-19: A systematic review and meta-analysis of clinical characteristics, risk factors, and outcomes. J Med Virol.

[12] Pande D, Kochhar A, Saini S, Ganapathy U, Gogia AR (2020). An update on initial epidemiological profile, clinical course, and outcome of COVID-19 patients at a tertiary care center in India. Indian J Palliat Care.

[13] Sepandi M, Taghdir M, Alimohamadi Y, Afrashteh S, Hosamirudsari H (2020). Factors associated with mortality in COVID19 patients: a systematic review and meta-analysis. Iran J Public Health.

[14] Perincek G, Avci S (2020). A case series of patients with COVID-19 infection admitted to a secondary care center in Turkey. Adv J Emerg Med.

[15] Macera M, De Angelis G, Sagnelli C, Coppola N, Vanvitelli Covid-Group (2020). Clinical presentation of COVID-19: case series and review of the literature. Int J Environ Res Public Health.

[16] Ahmed A, Ali A, Hasan S (2020). Comparison of epidemiological variations in COVID-19 patients inside and outside of China- A meta-analysis. Front. Public Health.

[17] Lim ZJ, Subramaniam A, Ponnapa Reddy M (2021). Case fatality rates for patients with COVID-19 requiring invasive mechanical ventilation A meta-analysis. Am J Respir Crit Care Med.

[18] Bonanad C, García-Blas S, Tarazona-Santabalbina F (2020). The effect of age on mortality in patients with covid-19: a meta-analysis with 611,583 subjects. J Am Med Dir Assoc.

[19] Tan L, Kang X, Ji X (2020). Validation of predictors of disease severity and outcomes in covid-19 patients: a descriptive and retrospective study. Med (NY).

[20] Xie J, Covassin N, Fan Z (2020). Association between hypoxemia and mortality in patients with COVID-19. Mayo Clin Proc.

[21] Kang SJ, Jung SI (2020). Age-related morbidity and mortality among patients with COVID-19. Infect Chemother.

[22] Cai R, Zhang J, Zhu Y (2021). Mortality in chronic kidney disease patients with COVID-19: a systematic review and meta-analysis. Int Urol Nephrol.

[23] Malek AE, Granwehr BP, Kontoyiannis DP (2020). Doxycycline as a potential partner of COVID-19 therapies. IDCases.

[24] Self WH, Semler MW, Leither LM (2020). Effect of hydroxychloroquine on clinical status at 14 Days in hospitalized patients with COVID-19: a randomized clinical trial. JAMA.

[25] Rajter JC, Sherman MS, Fatteh N (2021). Use of ivermectin is associated with lower mortality in hospitalized patients with coronavirus disease 2019: the ivermectin in COVID nineteen study. Chest.

[26] Deftereos SG, Giannopoulos G, Vrachatis DA (2020). Effect of colchicine vs standard care on cardiac and inflammatory biomarkers and clinical outcomes in patients hospitalized with coronavirus disease 2019: the GRECCO-19 randomized clinical trial. JAMA Netw Open.

[27] Zhao D, Zhang S, Igawa T, Frishman W (2020). Use of nonsteroidal anti-inflammatory drugs for covid-19 infection: adjunct therapy?. Cardiol Rev.

[28] Wong AY, MacKenna B, Morton CE (2021). Use of non-steroidal anti-inflammatory drugs and risk of death from COVID-19: an OpenSAFELY cohort analysis based on two cohorts. Ann Rheum Dis.

[29] Permana H, Huang I, Purwiga A (2021). In-hospital use of statins is associated with a reduced risk of mortality in coronavirus-2019 (COVID-19): systematic review and meta-analysis. Pharmacol Rep.

[30] Shrestha DB, Budhathoki P, Khadka S (2020). Favipiravir versus other antiviral or standard of care for COVID-19 treatment: a rapid systematic review and meta-analysis. Virol J.

[31] Spinner CD, Gottlieb RL, Criner GJ (2020). Effect of remdesivir vs standard care on clinical status at 11 days in patients with moderate COVID-19: A Randomized Clinical Trial. JAMA.

[32] Wang Y, Zhang D, Du G (2020). Remdesivir in adults with severe COVID-19: a randomised, double-blind, placebo-controlled, multicentre trial. Lancet.

[33] Beigel JH, Tomashek KM, Dodd LE (2020). Remdesivir for the Treatment of Covid-19 - Final Report. N Engl J Med.

[34] Kow CS, Merchant HA, Mustafa ZU, Hasan SS (2021). The association between the use of ivermectin and mortality in patients with COVID-19: a meta-analysis. Pharmacol Rep.

[35] Hariyanto TI, Halim DA, Jodhinata C, Yanto TA, Kurniawan A (2021). Colchicine treatment can improve outcomes of coronavirus disease 2019 (COVID-19): A systematic review and meta-analysis. Clin Exp Pharmacol Physiol.

[36] Ansarin K, Tolouian R, Ardalan M (2020). Effect of bromhexine on clinical outcomes and mortality in COVID-19 patients: A randomized clinical trial. Bioimpacts.

[37] Albani F, Fusina F, Granato E (2021). Corticosteroid treatment has no effect on hospital mortality in COVID-19 patients. Sci Rep.

[38] Sterne JAC, Murthy S, WHO Rapid Evidence Appraisal for COVID-19 Therapies (REACT) Working Group (2020). Association between administration of systemic corticosteroids and mortality among critically Ill patients with COVID-19: a meta-analysis. JAMA.

[39] Albani F, Fusina F, Giovannini A (2020). Impact of azithromycin and/or hydroxychloroquine on hospital mortality in COVID-19. J Clin Med.

[40] Arshad S, Kilgore P, Chaudhry ZS (2020). Henry ford COVID-19 task force Treatment with hydroxychloroquine, azithromycin, and combination in patients hospitalized with COVID-19. Int J Infect Dis.

[41] Permana H, Huang I, Purwiga A (2021). In-hospital use of statins is associated with a reduced risk of mortality in coronavirus-2019 (COVID-19): systematic review and meta-analysis. Pharmacol Rep.

[42] Bruce E, Barlow-Pay F, Short R (2020). Prior Routine Use of Non-Steroidal Anti-Inflammatory Drugs (NSAIDs) and Important Outcomes in Hospitalised Patients with COVID-19. J Clin Med.

[43] Wong AY, MacKenna B, Morton CE (2021). Use of non-steroidal anti-inflammatory drugs and risk of death from COVID-19: an OpenSAFELY cohort analysis based on two cohorts. Ann Rheum Dis.

[44] Chow JH, Khanna AK, Kethireddy S (2021). Aspirin use is associated with decreased mechanical ventilation, intensive care unit admission, and in-hospital mortality in hospitalized patients with coronavirus disease 2019. Anesth Analg.

[45] Pereira M, Dantas Damascena A, Galvão Azevedo et al (2020 ). Vitamin D deficiency aggravates COVID-19: systematic review and meta-analysis. Crit Rev Food Sci Nutr.

[46] Kamel AM, Sobhy M, Magdy N, Sabry N, Farid S (2021). Anticoagulation outcomes in hospitalized Covid-19 patients: A systematic review and meta-analysis of case-control and cohort studies. Rev Med Virol.

[47] Malek AE, Granwehr BP, Kontoyiannis DP (2020). Doxycycline as a potential partner of COVID-19 therapies. IDCases.

[48] Alam MM, Mahmud S, Rahman MM (2020). Clinical outcomes of early treatment with doxycycline for 89 high-risk COVID-19 patients in long-term care facilities in New York. Cureus.

